# HLA-A and HLA-DRB1 may play a unique role in ovarian teratoma-associated anti-N-methyl-D-aspartate receptor encephalitis

**DOI:** 10.1186/s12958-020-00661-5

**Published:** 2020-11-07

**Authors:** Xiaoya Zhao, Juan Li, Qian Zhu, Guiling Liang, Wei Xia, Xiaoqing He, Chenfeng Zhu, Hang Qi, Bo Deng, Xiangjun Chen, Jian Zhang

**Affiliations:** 1 Department of Obstetrics and Gynecology, International Peace Maternity and Child Health Hospital, School of Medicine, Shanghai Jiaotong University, No. 910, Hengshan Rd, Shanghai, 200030 China; 2 Department of Pathology, International Peace Maternity and Child Health Hospital, School of Medicine, Shanghai Jiaotong University, Shanghai, 200030 China; 3 Department of Neurology, Huashan Hospital and Institute of Neurology, Fudan University, No. 12 Wulumuqi Zhong Road, Shanghai, 200040 China

**Keywords:** Ovarian teratoma, Anti-N-methyl-D-aspartate receptor encephalitis, HLA-A, HLA-DRB1, Autoantibodies

## Abstract

**Background:**

Ovarian teratoma-associated anti-N-methyl-D-aspartate receptor encephalitis (NMDAR-E) is a severe autoimmune neurological disorder, and the influence of teratoma-induced autoantibodies on the pathogenesis remains unclear.

**Methods:**

Ovarian teratoma tissues were collected from teratoma patients with and without NMDAR-E. Proteins were extracted and then analyzed using iTRAQ-coupled LC–MS/MS, which was followed by bioinformatics analysis. Candidate proteins were verified by Western blotting and immunohistochemistry.

**Results:**

In total, 36 differentially expressed proteins (DEPs) were identified between the control group and NMDAR-E group, and the bioinformatics analysis revealed that the DEPs were mainly involved in immune-related pathways, especially HLA-A and HLA-DRB1. The western blotting results for HLA-A and HLA-DRB1 were consistent with the results of the iTRAQ analysis. Additionally, the immunohistochemical data revealed that the aggregation of HLA-A (+) and HLA-DRB1 (+) cells was more apparent in the teratoma tissues of NMDAR-E patients compared with that in the tissues of controls.

**Conclusion:**

Our investigation indicated that HLA-A and HLA-DRB1 might be involved in mediating ovarian teratoma-associated NMDAR-E. These findings provide new insights into the pathophysiological mechanisms and provide information for the functional exploration of proteins in the future.

## Background

Ovarian teratoma (OT) is one of the most common ovarian neoplasms, constituting 10–20% of all ovarian tumors in adults and almost half of all ovarian tumors in children. These tumors originate from three germ layers, namely, the ectoderm, mesoderm, and endoderm [1, 2]. However, women with teratomas might also suffer from anti-N-methyl-D-aspartate receptor encephalitis (NMDAR-E), a paraneoplastic syndrome that typically results in progressive neuropsychiatric symptoms, hyperkinetic movements, dysautonomia, seizures and autoantibodies against the central nervous system (CNS) [3–7]. NMDAR-E is a recently described severe autoimmune neurological disorder with a progressive clinical course caused by specific IgG antibodies targeting the GluN1 subunit of NMDAR in patient cerebrospinal fluid (CSF) [3, 8]. Unfortunately, the diagnosis of OT-associated NMDAR-E is time consuming and difficult. Patients are examined initially by neurologists or psychiatrists due to developing psychiatric symptoms that are misdiagnosed as mental illness and are forced to take antipsychotic drugs, which not only have side effects but also delay the cure by allowing the illness to progress to dyspnea or even death [5, 9]. Once the teratoma is discovered in NMDAR-E patients, timely surgery to remove the teratoma may be beneficial for the long-term prognosis, which suggests that the production of autoantibodies is associated with the teratoma [4, 5, 7, 10, 11].

To date, most studies have focused more on neurology or psychiatry rather than gynecology, and most have explored the etiology and pathogenesis of OT-associated NMDAR-E [9] by analyzing the biological and molecular differences in CSF and serum between teratoma patients with and without NMDAR-E [12–14]. Mueller et al. reported only a borderline association of HLA-B*07:0 with NMDAR-E in German patients [15]. Shu et al. reported that the HLA class II allele DRB1*16:02 was associated with an increased risk of NMDAR-E in the Chinese Han population [16]. These allele studies highlight the possible association between HLA alleles and NMDAR-E and note that T cells may be involved in pathogenesis [17].

To our knowledge, there are currently few studies focusing on teratoma tissue. Furthermore, the mechanism of teratoma tissue causing NMDA-R have different opinions. Earlier histopathological studies suggested that neuroglial elements within teratomas may be integral to autoantibody production, disease pathogenesis, and neuronal morphological changes in teratomas associated with NMDAR-E [3]. In contrast, later studies found that the presence of neuroglial tissues within teratomas is not sufficient to activate the anti-NMDAR autoimmune response, suggesting that OT-associated NMDAR-E in patients may be tumor-specific [18–20]. In addition to the study of teratomas on the autoimmunity, several studies demonstrated that teratomas exhibit differences in inflammatory infiltration in patients with and without NMDAR-E [21, 22].

Based on previous studies, OT-associated NMDAR-E is most likely an autoimmune inflammatory disorder affecting the CNS [23, 24]. However, the lack of studies on teratoma tissue in NMDAR-E patients has resulted in incomplete insights into the potential association between teratoma and NMDAR-E. Furthermore, some limitations still exist, including the incomplete understanding of the true molecular mechanisms responsible for OT-associated NMDAR-E, and the teratoma tissues that cause NMDAR-E remain unclear. Consequently, we conducted proteomic studies that identify the abnormally expressed proteins in the teratoma tissue of NMDAR-E patients and provide clues for exploring the pathophysiological mechanisms at the protein level in the future.

## Method

### Patients and samples

A control group of eight Chinese women who were diagnosed with mature ovarian teratoma without NMDAR-E was formed (C-01, C-02, C-03, C-04, C-05, C-06, C-07, and C-08). The electronic medical records were reviewed to confirm that none of the patients in the control group had concurrent encephalitis. The NMDAR-E group was composed of four Chinese women who were diagnosed with mature ovarian teratoma with NMDAR-E: S-01, S-02, S-03, and S-04. The criteria for the diagnosis of NMDAR-E were based on the clinical evaluation by the treating neurologist as documented in the electronic medical record and based on the presence of stereotypical symptoms and progression over time. The clinical diagnosis was confirmed by a positive result for testing of the anti-NMDAR antibody titer in the CSF and/or serum in the NMDAR-E group. C-01 to C-04 and S-01 to S-04 were subjected to iTRAQ-based quantitative proteomic analysis and western blotting, and C-01 to C-08 and S-01 to S-04 were subjected to immunohistochemistry (IHC). Teratoma tissues were collected at the Department of Obstetrics and Gynecology of the International Peace Maternity and Child Health Hospital (IPMCH), Shanghai, between September 2018 and December 2019. The 0.5–1.0 cm samples were obtained immediately after surgical resection of the teratoma, and each tissue sample was rinsed twice with ice-cold PBS to remove residual blood cells and then frozen in liquid nitrogen within 1 min. Then, the tissues were transported from the operating room to the laboratory within 10 min. The formalin-fixed, paraffin-embedded teratoma tissues used for IHC were collected at the Department of Pathology, IPMCH. All clinical samples were obtained with the written informed consent of all participants, and this study was approved by the institutional ethics committee of the International Peace Maternity and Child Health Hospital in Shanghai, China (GKLW-2018-41). All experiments were performed in accordance with the guidelines and regulations of the International Peace Maternity and Child Health Hospital.

### Protein extraction

Protein was extracted by the acetone precipitation method according to the protocol of the Beijing Genomics Institute (BGI) (Wuhan, China). The samples were transferred into a 2 ml centrifuge tube, and two steel beads and 1X cocktail with appropriate amounts of SDS L3 and EDTA were added. Then, the mixtures were placed on ice for 5 min, and 10 mM dithiothreitol (DTT) was added; the mixtures were then placed into a grinder for 2 min at 60 Hz and centrifuged at 25,000 g for 15 min at 4 °C. The supernatant was incubated with 10 mM DTT at 56 °C for 1 h and alkylated with 55 mM iodoacetamide (IAM) in the dark at room temperature for 45 min. The solution was added to cold acetone at a ratio of 1:5, and the mixture was placed in a refrigerator at − 20 °C for 30 min and then centrifuged at 25,000 g for 15 min at 4 °C; the supernatant was discarded. Subsequently, the protein was precipitated by air drying, and lysis buffer without SDS L3 was added; a grinder was used for 2 min at 60 Hz to promote protein solubilization. After centrifugation at 25000 g for 15 min at 4 °C, the protein in the supernatant was quantified by the Bradford method.

### Protein digestion

Protein samples (100 μg) were diluted with 0.5 M TEAB to bring the final concentration of urea to below 2 M, and the final concentration of sodium dodecyl sulfate (SDS) was less than 0.1%. Trypsin Gold (Promega, Madison, WI, USA) was used to digest the proteins with a protein:trypsin ratio of 20:1. The enzyme solution was added, vortexed, centrifuged at low speed for 1 min, and incubated at 37 °C for 2 h. After trypsin digestion, the peptides were desalted and freeze-dried according to the manufacturer’s protocol.

### Peptide labeling

The peptides were reconstituted in 0.5 mmol/L TEAB and mixed with 50 μl of isopropanol. The samples were labeled with the iTRAQ Reagent 8-plex Kit according to the manufacturer’s protocol (AB Sciex, Foster City, CA, USA). The peptides labeled with different reagents were combined, desalted and freeze-dried.

### Peptide fractionation and LC-MS/MS analysis

The peptides were separated on a Shimadzu LC-20AB HPLC pump system coupled with a high pH RP column. The peptides were reconstituted with buffer A (5% ACN pH 9.8). The peptides were eluted at a flow rate of 1 mL/min with a series of gradients of buffer B: 5% buffer B (95% ACN, pH 9.8) for 10 min, 5–35% buffer B for 40 min, 35–95% buffer B for 1 min, and 95% buffer B for 3 min. The column was then equilibrated with 5% buffer B for 10 min. The elution was monitored by measuring the absorbance at 214 nm, and fractions were collected every 1 min. The eluted peptides were pooled into 20 fractions and freeze-dried.

After the above steps were performed, the dried peptide samples were resuspended in buffer A (2% ACN, 0.1% FA) and centrifuged at 20000 g for 10 min. The supernatant was loaded onto a C18 trap column at 5 μL/min for 8 min using a nano-HPLC instrument (LC-20 AD, Shimadzu, Kyoto, Japan) with an autosampler. Then, the peptides were eluted from the trap column and separated by an analytical C18 column (inner diameter of 75 μm) that was packed in-house. The gradient was run at 300 nL/min starting with 5% of buffer B (98% ACN, 0.1% FA) for 5 min, which was increased from 5 to 25% over 5 min, 25–35% over 45 min, and 35–80% over 50 min, and then it was maintained at 80% over 54 min, finally returned to 5% for 0.1 min and allowed to equilibrate for 60 min.

The peptides separated with nano HPLC were subjected to tandem mass spectrometry with a Q Exactive mass spectrophotometer (Thermo Fisher Scientific, San Jose, CA, USA) for data-dependent acquisition (DDA) detection by nanoelectrospray ionization. The main parameters were as follows: electrospray voltage: 1.9 kV; MS1 scan range: 350–1500 m/z at a resolution of 60,000 in the Orbitrap; the MS2 starting m/z was fixed at 100 m/z at a resolution of 15,000 in HCD mode; dynamic exclusion time: 15 s; automatic gain control (AGC) for the full MS target and the MS2 target: 3 × 10^6^ and 1 × 10^5^, respectively. The ion screening conditions for MS2 fragmentation were as follows: charge 2+ to 6+, and the top 20 parent ions had a peak intensity exceeding 10,000.

### Protein quantification and iTRAQ data analysis

An automated software called IQuant [25] was used to quantitatively analyze the labeled peptides with isobaric tags, and it integrates Mascot Percolator to provide reliable significance measures. Subsequently, the PSMs were prefiltered according to a false discovery rate (FDR) of 1% to assess the confidence of the peptide identifications. Based on the picked protein FDR strategy [26], a protein FDR of 1% will also be utilized after protein inference (protein-level FDR ≤ 0.01) to control the rate of false positives at the protein level.

The protein quantification process includes the following steps: protein identification, tag impurity correction, data normalization, missing value imputation, protein ratio calculation, statistical analysis, and results presentation.

The raw data were analyzed by the Thermo Scientific tool Proteome Discoverer and searched using Mascot version 2.3.02 in this project. The results were aligned to the canonical proteomics database to obtain the final protein identification results.

### Bioinformatics

The raw MS/MS data were converted into MGF format, and the exported MGF files were searched by the local Mascot server against the database described above. In addition, quality control was performed to determine if a reanalysis step was needed. IQuant [25] was applied to the quantification of proteins. A fold change ≥2 and a *P* value < 0.05 were considered to indicate the differentially expressed proteins (DEPs). Furthermore, more in-depth analyses based on the DEPs, including GO enrichment analysis, KEGG pathway enrichment analysis, Eukaryotic Orthologous Groups (KOG) functional annotation, cluster analysis, protein interaction analysis and subcellular localization analysis, were also performed.

### Western blotting analysis

To validate the accuracy of the iTRAQ results, western blotting analysis was performed to examine the relative abundances of some of the differentially expressed proteins. Proteins extracted from the control group and NMDAR-E group were separated by SDS-PAGE and then electrotransferred to a polyvinylidene fluoride (PVDF) membrane. The membranes were blocked for 2 h at room temperature in PBST containing 5% skim milk and then incubated with rabbit monoclonal anti-HLA Class II DRB1 antibody (1:1000, ab133578, Abcam, UK) and rabbit monoclonal anti-HLA A A antibody (1:1000, ab52922, Abcam, UK) overnight at 4 °C. The membranes were incubated with secondary antibodies for 1 h at room temperature after washing them three times with PBST. The bands were visualized using enhanced chemiluminescence (ECL) with an ImageQuant LAS 4000 detection system (GE, Uppsala, Sweden), and the proteins were quantified using Fiji software [27].

### Immunohistochemistry (IHC)

Briefly, formalin-fixed, paraffin-embedded samples were obtained from the Department of Pathology, IPMCH, from patients C-01 to C-08 and S-01 to C-04. The tumor regions were reviewed and assessed by a pathologist. With a microtome, 5 μm sections from the tissue blocks were obtained. Rabbit polyclonal antibodies against HLA Class II DRB1 (HLA-DRB1) (1:2000, ab133578, Abcam, UK) and HLA Class I A (HLA-A) (1:400, ab52922, Abcam, UK) were used as the primary antibodies. IHC assays for HLA-A and HLA-DRB1 were performed by RecordBio (Shanghai, China). Sections were deparaffinized, rehydrated and processed for antigen retrieval by a standard microwave heating technique in antigen retrieval solution (pH 8). Endogenous peroxidase activity was quenched by incubating the sections in 3% H2O2 for 25 min. The sections were blocked with 3% bovine serum albumin at room temperature for 30 min and were then incubated overnight with the primary antibody at 4 °C, which was followed by incubation with the secondary antibody (ready to use, PV-1001, ZSGB-BIO) at room temperature for 50 min. Slides were visualized using diaminobenzidine tetrahydrochloride, and nuclei were counterstained with hematoxylin. Each section was randomly assessed based on 10 high-power fields (× 400). The expression of HLA-A and HLA-DRB1 was assessed by semiquantitative scoring of the staining intensity (scale 0–3) and the percentage of positive cells (0–100%). The staining intensity and positive cell scores were then multiplied, generating a score ranging from 0 to 300. To maintain the consistency of the measurements, the same qualified pathologist assessed each sample and analyzed/scored the IHC data with Image-Pro Plus 6.0 [28].

### Statistical analysis

Student’s t-test or a Mann-Whitney U test was used to determine the statistical significance of the difference between the control group and NMDAR-E group. Continuous variables are presented as the mean ± standard deviation (SD). Significant differences were assessed using GraphPad Prism 8 (GraphPad Software Inc., La Jolla, CA, USA), and *P* < 0.05 was considered statistically significant. The bioinformatic analysis was performed by Beijing Genomics Institute (BGI) (Wuhan, China). Significant differential expression was indicated by a |log2 (fold change)| ≥ 1.0 and a *p* value< 0.05, which were considered statistically significant.

## Results

### Clinical information

The clinical characteristics of all participants are summarized in Table 1. No significant differences were found in terms of group homogeneity between the patients with teratomas with and without NMDAR. The workflow of this study was as follows: proteins were extracted from the collected samples, which was followed by quality control and proteomic analysis of eight samples using iTRAQ quantification proteomics. Subsequently, the DEPs were analyzed by bioinformatics analysis to determine the key pathways that play an important role in the pathogenesis of OT-associated NMDAR-E. Finally, the candidate proteins HLA-A and HLA-DRB1 were selected and further confirmed by western blotting and IHC.

**Table 1:** Demographics and clinical characteristics for the experiment and validation samples

Sample number	Age (years)	psychotic disorders	Endometriosis	Pelvic inflammatory disease	Teratoma	NMDAR-E	Anti-NMDAR antibody	
							Cerebrospinal fluid	Serum
S-01	28	√	×	×	right, mature	√	**+**	**+**
S-02	17	√	×	×	left, mature	√	**+**	**+**
S-03	14	√	×	×	right, mature	√	**+**	**+**
S-04	19	√	×	×	bilateral, mature	√	**+**	**+**
C-01	23	×	×	×	left, mature	×	**–**	**–**
C-02	19	×	×	×	right, mature	×	**–**	**–**
C-03	21	×	×	×	right, mature	×	**–**	**–**
C-04	27	×	×	×	right, mature	×	**–**	**–**
C-05	23	×	×	×	right, mature	×	**–**	**–**
C-06	24	×	×	×	left, mature	×	**–**	**–**
C-07	27	×	×	×	bilateral, mature	×	**–**	**–**
C-08	21	×	×	×	left, mature	×	**–**	**–**

### Identification of proteins by iTRAQ quantitative proteomics analysis

**Fig. 1:**
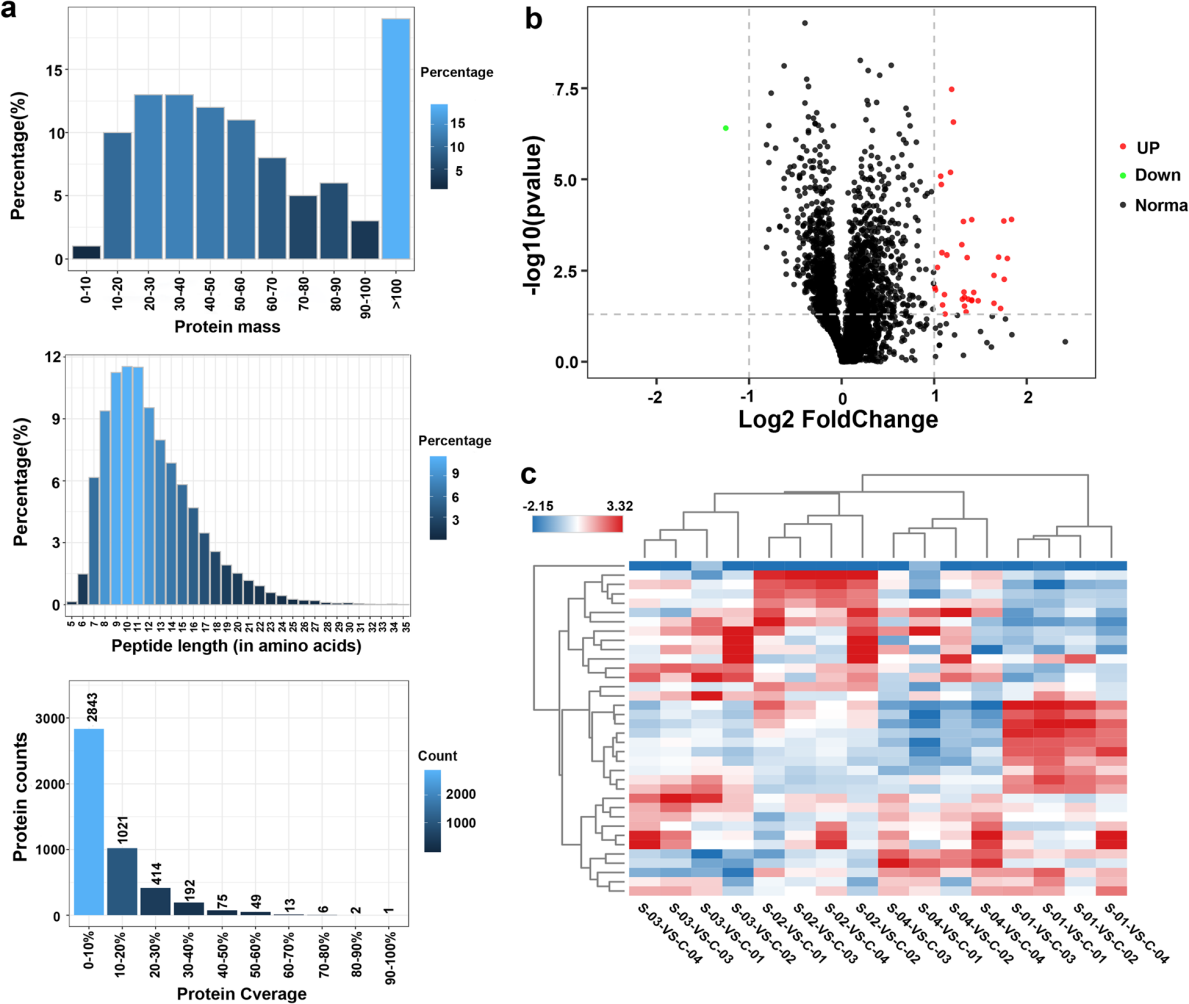
The teratoma tissue proteome dataset from the iTRAQ quantitative proteomics analysis. **a** Protein mass distribution (X-axis: Molecular weight (kDa); Y-axis: Percentage of protein number). Peptide length distribution (X-axis: peptide length; Y-axis: corresponding peptide). Protein coverage distribution (X-axis: protein coverage range; Y-axis: corresponding protein number). **b** The volcano plot of the differentially expressed genes identified by iTRAQ analysis. The differentially expressed proteins, including 35 upregulated proteins and 1 downregulated protein, are illustrated in a volcano plot (|log2 fold change | ≥ 1.0 & *P* < 0.05). Each point (dot) represents the gene expression value; green (downregulated in the NMDAR-E group) and red (upregulated in the NMDAR-E group) dots represent significant DEPs. Black dots indicate nonsignificant proteins. **c** Cluster analysis of gene expression profiles. The color bar indicates the log2 fold change range between upregulation (red) and downregulation (blue)

The four samples in the NMDAR-E group included S-01, S-02, S-03, and S-04, and the four samples in the control group included C-01, C-02, C-03, and C-04. To reduce interindividual variation, the samples were pooled together and analyzed by iTRAQ quantitative proteomics. In total, 853,152 spectra were generated, and 18,790 peptides and 4608 proteins were identified with a 1% FDR. Among all the identified proteins, 50% of the proteins had a molecular weight (kDa) of approximately 10–50 kDa, while 18% of the proteins had a molecular weight (kDa) over 100 kDa. The length of approximately 50% peptides was 5–11 amino acids, and the sequence coverage of over 90% of proteins was less than 30% (Fig. 1a).

### Differentially expressed protein identification

Based on the cutoff values of a |log2 fold change| ≥1.0 and a *P* value < 0.05, 36 out of the 4608 proteins were found to be significantly differentially expressed in the NMDAR-E group compared with the control group, including 35 upregulated proteins and 1 downregulated protein (Supplementary Table 1). The *p* values and log2 fold change values of the DEPs are visualized in the volcano plot (Fig. 1b). The expression profiles of the DEPs were determined by hierarchical clustering to more intuitively identify the differences between the NMDAR-E group and the control group (Fig. 1c).

### Classification of differentially expressed proteins

**Fig. 2:**
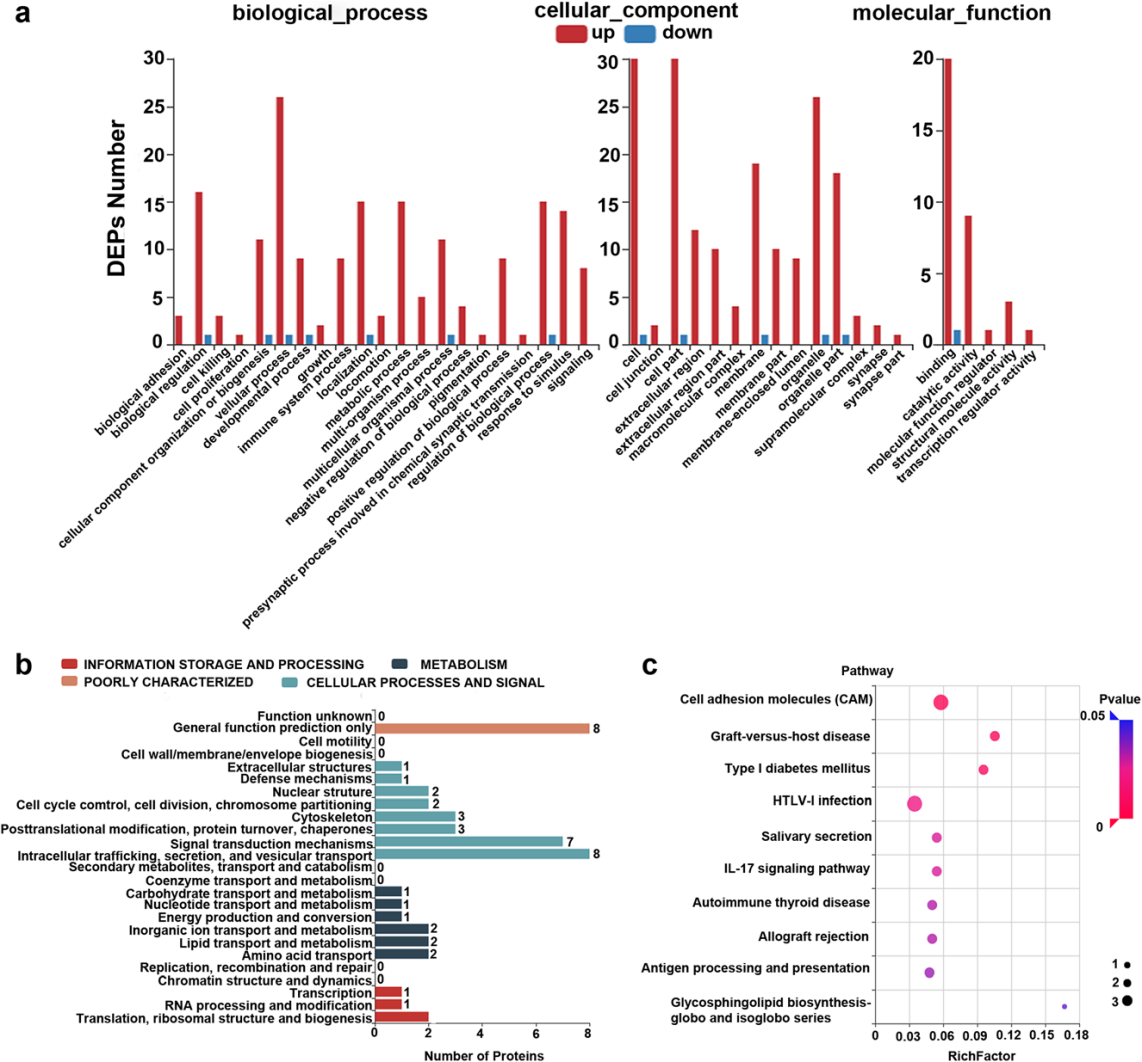
Functional classifications of differentially expressed proteins. **a** The enriched GO terms according to the values of the enrichment score for BP, MF and CC categories (X-axis: GO terms; Y-axis: number of target genes for each term). **b** KOG enrichment analysis of the DEPs (x-axis: number of target genes for each term; y-axis: KOG terms). (c) KEGG pathway analysis of the DEPs (|log2 fold change | ≥ 1.0 & *P* < 0.05) shown as a scatter plot. Enrichment ratio: the ratio of the number of target genes in each pathway. The size represents the number of enriched genes. The *P* value, ranging from 0 to 1, is shown according to the color

A total of 36 DEPs were subjected to integrated GO, KEGG, and KOG enrichment analyses to further understand the possible physiological processes and pathways. GO enrichment analysis indicated that 40 GO terms were enriched with a *p* value < 0.05, and the values of the enrichment scores for biological processes (BP), molecular functions (MF) and cellular components (CC) are illustrated in Fig. 2a. In the NMDAR-E group compared to the control group, the DEPs in the BP category were mainly distributed in immune system processes, cellular processes, biological regulation, and metabolic processes. In the CC category, the proteins were located in the membrane, extracellular region, cell, and cell part. The binding, catalytic activity, and structural molecular activity were mainly enriched in the MF category.

To better identify orthologous and paralogous proteins, the DEPs were analyzed by KOG analysis. Based on the KOG analysis, the DEPs were classified into four functional categories: information storage and processing, metabolism, cellular process and signaling, and poorly characterized (Fig. 2b). KEGG enrichment analysis was employed to discover how proteins participate in the biological processes related to OT-associated NMDAR-E (Fig. 2c and Supplementary Fig. 1). KEGG analysis revealed that HLA class I histocompatibility antigen A (HLA-A) and HLA class II histocompatibility antigen DRB1 (HLA-DRB1) participated in immune-related biological processes such as antigen processing and presentation, graft-versus-host disease, allograft rejection, and autoimmune thyroid disease, which may play an important role in the pathways of immune diseases.

Protein targeting or sorting is a biological mechanism by which proteins are transported to their correct destinations inside or outside the cell. Based on the protein information, proteins can be targeted to the inner space of an organelle, different intracellular membranes, the plasma membrane, or to the exterior of the cell via secretion. DEP subcellular localization prediction was performed by WoLF PSORT [29] and is shown in Supplementary Fig. 2.

### Protein-protein interaction analysis

**Fig. 3:**
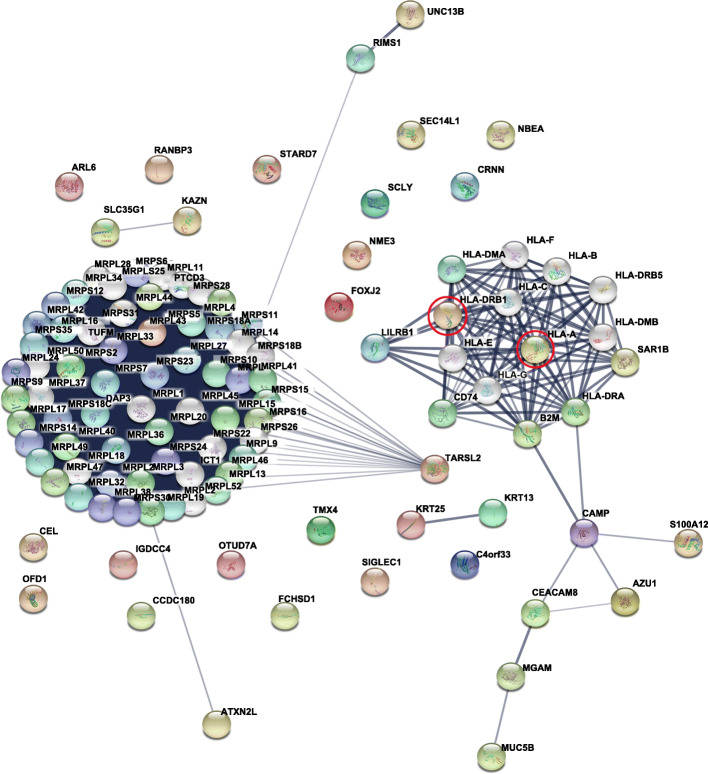
Protein-protein interaction network of 36 proteins differentially expressed between the control group and the NMDAR-E group. Proteins selected for further western blot validation are indicated with red circles

Among the 36 differentially expressed proteins, a protein-protein interaction (PPI) network was constructed by STRING [30] to identify various possible interactions, including direct physical and indirect functional associations, which provided a potential analysis platform for further study of the molecular mechanism based on the complex interactions among DEPs. In the present study, the PPI network was mainly divided into two categories. One of the central areas comprised proteins related to HLA-A and HLA-DRB1 in the NMDAR-E group, which revealed that the two proteins interacted with each other and were functionally associated with immune-related proteins, such as CD80 and another human leukocyte antigen (HLA) subtype (Fig. 3 and Supplementary Fig. 3).

### Validation of differentially expressed proteins critical to OT-associated NMDAR-E

**Fig. 4:**
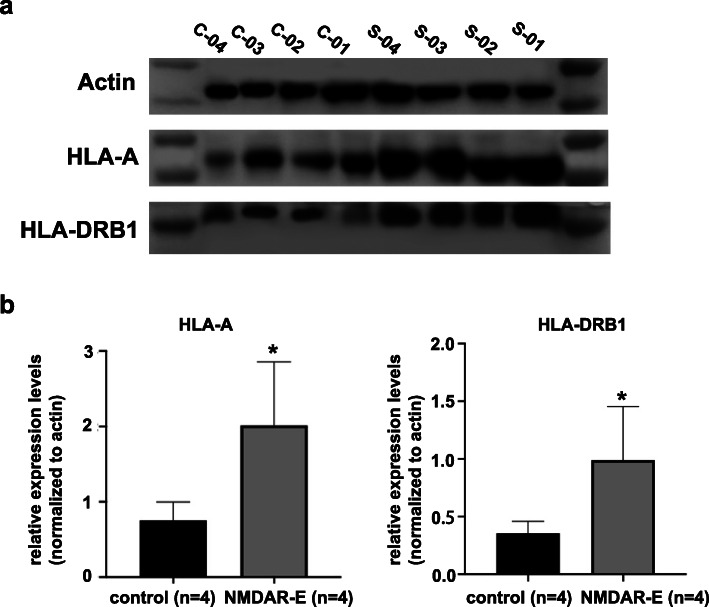
Western blotting validation of differentially expressed proteins. **a** Verification of the expression of HLA-A and HLA-DRB1 in the control group and NMDAR-E group by western blotting analysis. The full-length blots are presented in Supplementary Fig. 4. **b** Quantitative assessment of protein expression using densitometric analysis. Four teratoma patients without NMDAR-E were in the control group (C-01 to 04), and 4 teratoma patients with NMDAR-E were in the NMDAR-E group (S-01 to 04). Data are represented as the mean ± SD. *P < .05

Based on the above bioinformatics analysis results, HLA-A and HLA-DRB1 were shown to be involved in various immune-related molecules and pathways, which probably participated in the pathogenesis of encephalitis. To verify that the expression of HLA-A and HLA-DRB1 were higher in teratoma patients with NMDAR-E than in teratoma patients without NMDAR-E, western blotting and IHC were performed on the teratoma tissue with and without NMDAR-E to identify the differences in HLA-A and HLA-DRB1 expression. The results of western blotting analysis revealed that HLA-A and HLA-DRB1 were indeed highly expressed in the patients with OT-associated NMDAR-E (Fig. 4a-b and Supplementary Fig. 4), which was consistent with the results achieved through iTRAQ analysis.

**Fig. 5:**
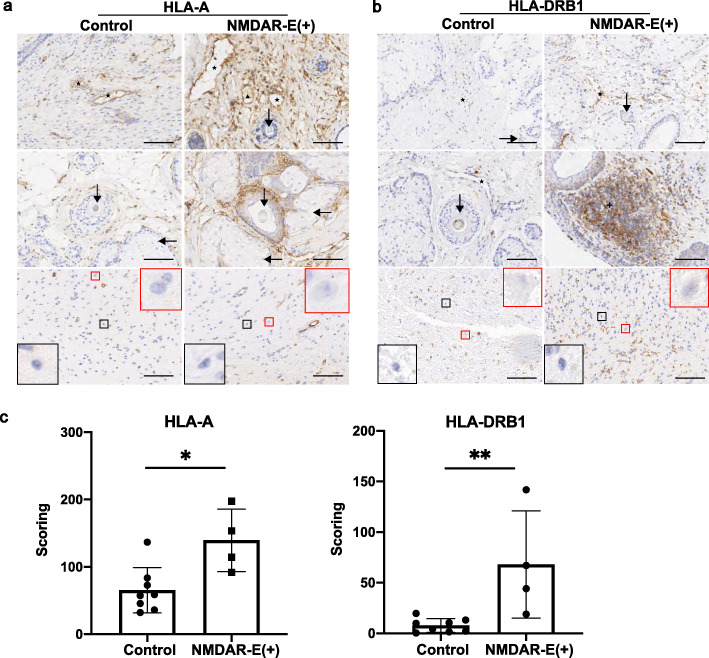
Immunohistochemical findings in the teratoma tissues of the NMDAR-E group and the control group. **a** HLA-A (+) cells were more densely aggregated in the teratoma tissues in the NMDAR-E (+) patients in comparison with those in the controls. **b** HLA-DRB1 (+) cells were evident in the teratoma tissues in the NMDAR-E (+) patients but were scarcely detected in the controls. **c** The IHC score values of HLA-A and HLA-DRB1 were significantly higher in the teratoma tissues of NMDAR-E (+) patients compared with those in tissues of controls. There were eight teratoma patients without NMDAR-E in the control group (C-01 to 08) and 4 teratoma patients with NMDAR-E in the NMDAR-E group (S-01 to 04). Data are represented as the mean ± SD. *P < .05, ** *P* < .01. bar = 100 μm. (★: blood vessels, ▲: lymphatic vessels, ✚: germinal center, ↓: hair follicle, ←: sebaceous glands, red square: neuron, black square: glial cells)

The IHC data for teratoma tissue staining in the four teratomas with NMDAR-E and the eight teratomas without NMDAR-E is shown. The results showed that the numbers of HLA-A (+) and HLA-DRB1 (+) cells in the teratoma tissues were significantly larger in the NMDAR-E (+) patients than those in the controls (Fig. 5a-b). The IHC score values of HLA-A and HLA-DRB1 were significantly higher in the NMDAR-E (+) patients than in the controls (Fig. 5c), which is consistent with the iTRAQ results.

## Discussion

OT-associated NMDAR-E is a serious and potentially fatal autoimmune synaptic encephalitis that commonly occurs in young women, and gynecologists often lack relevant experience with the disease [9, 31, 32]. Although the exact incidence of OT-associated NMDAR-E is still unknown, many cases have been identified around the world, which indicates that OT-associated NMDAR-E is much more common than previously believed [7, 33–35]. Furthermore, many patients with OT-associated NMDAR-E not only suffer from disease pain and a certain degree of family financial burden but also show long-term effects or even incomplete recovery. Previous studies indicated that the abnormal neuronal elements within teratomas may be essential in triggering the breakdown of immune tolerance and promoting the development of autoimmunity in OT-associated NMDAR-E [3, 18, 20, 22, 36]. However, it is noteworthy that the presence of a teratoma containing neuroglial tissue is not sufficient to induce NMDAR-E [18–20]. In addition, OT-associated NMDAR-E was associated with increased lymphocyte infiltration in the teratoma tissues [18, 20–22]. However, the regulatory role of teratoma tissues in the pathogenesis and etiology of NMDAR-E remains unknown.

In the present study, we first profiled protein expression using the iTRAQ-coupled LC-MS/MS method to identify 36 unique DEPs, which was followed by GO enrichment, KEGG pathway annotation, KOG function annotation, subcellular localization prediction and PPI analyses to identify the representative DEPs HLA-A and HLA-DBR1, which are involved in immune-related and inflammatory pathways and interact with other immune proteins. Subsequently, the candidate DEPs (HLA-A and HLA-DBR1) were further confirmed by western blotting and IHC. In patients with OT-associated NMDAR-E, the levels of HLA-A and HLA-DBR1 expression were significantly higher, and aggregates of HLA-A (+) and HLA DRB1 (+) cells were apparent in the teratoma tissues, which was in accordance with the iTRAQ-LC-MS/MS analysis.

HLA molecules are essential to regulate immune surveillance and responsiveness in health and disease [37]. In particular, the immunological manifestations of the majority of diseases are consistent with the enrichment of dominant immune genes within the major histocompatibility complex (MHC) region. The three subclasses in the MHC region include HLA class I, HLA class II, and HLA class III [37]. The HLA-A protein, which is a single-pass transmembrane protein, represents one of three major subclasses of HLA class I cell surface receptors, which form a complex with B2 M/beta2 microglobulin that binds viral and tumor-derived peptides for presentation to HLA-A–restricted CD8+ T cells to guide the acquired immune response to eliminate aberrant cells [38–40]. The HLA-DRB1 protein encoded by the HLA-DRB1 gene belongs to the beta chain of the HLA class II molecule and is anchored in the membrane. It plays a crucial role in the immune response by presenting antigenic peptides to HLA-DRB1-restricted CD4+ T cells [41, 42]. Furthermore, the key role of HLA-A-restricted CD8+ T cell responses is initiating autoimmunity and interactions with HLA class II-restricted CD4+ T cell responses [43, 44]. The NMDAR antibody immune response was more relevant than CD8+ T cell mechanisms to the pathogenesis of NMDAR-E [22]. Upregulation of HLA class I and class II receptors along with alterations in CD80 and CD86 expression on antigen presenting cells (APCs) can trigger the breakdown of T cell tolerance mechanisms and generate autoimmune antibodies [45]. Based on previous studies, various HLA-A and HLA-DRB1 alleles have been shown to be associated with increased susceptibility to multiple autoimmune diseases, such as anti-LGI1 encephalitis, multiple sclerosis (MS), and rheumatoid arthritis (RA) [43, 44, 46–48]. In the Shu et al. study, the blood samples of 61 patients with NMDAR-E and 571 healthy controls from the Chinese Han population were collected and genotyped by a PCR sequencing-based typing method, which indicated that the HLA class II allele DRB1*16:02 increased susceptibility to NMDAR-E [16, 17]. Notably, autoimmune diseases associated with the DRB1*16:02 allele were mainly mediated by autoantibodies, which indicated that the DRB1*16:02 allele was potentially involved in autoantibody generation [16].

As far as we know, CD19, CD20, and MHC class II antigens are first expressed at the precursor B cell stage and increase at the immature B cell stage. The membrane surface of mature B cells expresses mIgD to prevent immune tolerance, and B cells are activated with the assistance of CD4+ T cells and differentiate into plasma cells to synthesize and secrete immunoglobulin proteins. An immunohistochemical study of teratomas showed that the frequency and mode differed markedly between patients with and without NMDAR-E for CD4+ T cells, CD8+ T cells, and CD20+ B cells; in particular, increased CD20+ B lymphocyte infiltration around neuroglial tissues was characteristically observed in OT-associated NMDAR-E patients [18, 20–22]. In the autoimmune disease MS, Jelcic et al. reported that the interaction of B and T lymphocytes probably plays important roles in the autoimmune response [49]. Additionally, it has been proposed that the presence of a teratoma leads to the production of unknown autoantigens, resulting in the expansion of B and T lymphocytes and tumor-specific antibodies and ultimately leading to cross-reactivity with NMDARs [50].

Based on the above information, several hypotheses can be proposed to explain the autoimmune response in OT-associated NMDAR-E patients. 1) The interaction of B and T cells may play a certain role in the disease. 2) The susceptibility to NMDAR-E, which is associated not only with HLA molecules but also with inherited deficits of the innate immune system, may result in the generation of autoimmunity and disruption of the blood-brain barrier (BBB), leading to the development of a broad range of psychiatric symptoms. 3) An unknown self-antigen produced in teratoma tissues leads to increases in the HLA-A and HLA-DRB1 proteins, producing autoantibodies that pass through the blood-brain barrier and attack the central nervous system. However, there is currently no direct evidence to prove this hypothesis.

Several limitations of the present study should be mentioned here. First, the sample size is relatively small as a result of difficulties in collecting OT-associated NMDAR-E samples. In addition, there is a lack of functional research studies and mechanistic exploration on the topic of OT-associated NMDAR-E. Further studies are needed to validate the proteins and pathways identified in this study before drawing definitive conclusions.

## Conclusion

In summary, our investigation, by utilizing iTRAQ-based quantitative analysis and bioinformatics methods, first constructed a proteomics map for individuals with teratomas with and without NMDAR-E to increase the understanding of the underlying mechanism of NMDAR-E at the protein level. The present study may contribute to deeper insights into the pathophysiological mechanisms and provide clues for the subsequent search for biomarkers of OT-associated NMDAR-E. In the future, we will further expand the sample size for verification and carry out functional experiments to explore the pathological mechanism of OT-associated NMDAR-E. Furthermore, we will detect proteins, including HLA-A and HLA-DRB1, and determine the percentage of B lymphocytes in peripheral blood through flow cytometry with the goals of finding ideal biomarkers for early clinical screening, early diagnosis and prognosis prediction as well as optimizing the strategies used for diagnosis and treatment.

## Supplementary Information

**Additional file 1: Supplementary Table S1.** The differentially expressed proteins in iTRAQ.

**Additional file 2: Supplementary Figure S1.** The statistics of differentially expressed proteins by KEGG Pathway.

**Additional file 3: Supplementary Figure S2.** Subcellular localization prediction (x-axis: subcellular structure; y-axis: protein count.) analysis of differentially expressed proteins.

**Additional file 4: Supplementary Figure S3.** Protein-protein interaction network of 36 differentially expressed proteins were subdivided into two categories: the network of colorful dot consisted of immune-related proteins.

**Additional file 5: Supplementary Figure S4.** Full-length western blots for Fig. 4a.

## Data Availability

The data generated during the current study are available from the corresponding author on reasonable request.

## Abbreviations

OT: Ovarian teratoma
NMDAR-E: Anti-N-methyl-D-aspartate receptor encephalitis
iTRAQ: Isobaric tag for relative and absolute quantitation
LC-MS: Liquid chromatography-mass spectrometry
CSF: Cerebrospinal fluid
CNS: Central nervous system
DTT: Dithiothreitol
IAM: Iodoacetamide
SDS: Sodium dodecyl sulfate
DDA: Data-dependent acquisition
AGC: Automatic gain control
FDR: False discovery rate
DEPs: Differentially expressed proteins
KOG: Eukaryotic Orthologous Groups
PVDE: Polyvinylidene fluoride
ECL: Enhanced chemiluminescence
IHC: Immunohistochemistry
SD: Standard deviation
BGI: Beijing Genomics Institute
BP: Biological process
MF: Molecular functional
CC: Cellular component
HLA: Human leukocyte antigen
HLA-A: HLA class I histocompatibility antigen, A
HLA-DRB1: HLA class II histocompatibility antigen, DRB1
MHC: Major histocompatibility complex
APCs: Antigen presenting cells
MS: Multiple sclerosis
RA: Rheumatoid arthritis
BBB: Blood-brain barrier
