# Supplementary material for: HLA-A and HLA-DRB1 may play a unique role in ovarian teratoma-associated anti-N-methyl-D-aspartate receptor encephalitis
Source: Reprod Biol Endocrinol. 2020 Nov 7;18:107. doi: 10.1186/s12958-020-00661-5 (PMC7648266; doi:10.1186/s12958-020-00661-5)
Supplement: Supplementary file 2 — Additional file 2: Supplementary Figure S1. The statistics of differentially expressed proteins by KEGG Pathway. [file 12958_2020_661_MOESM2_ESM.pdf]

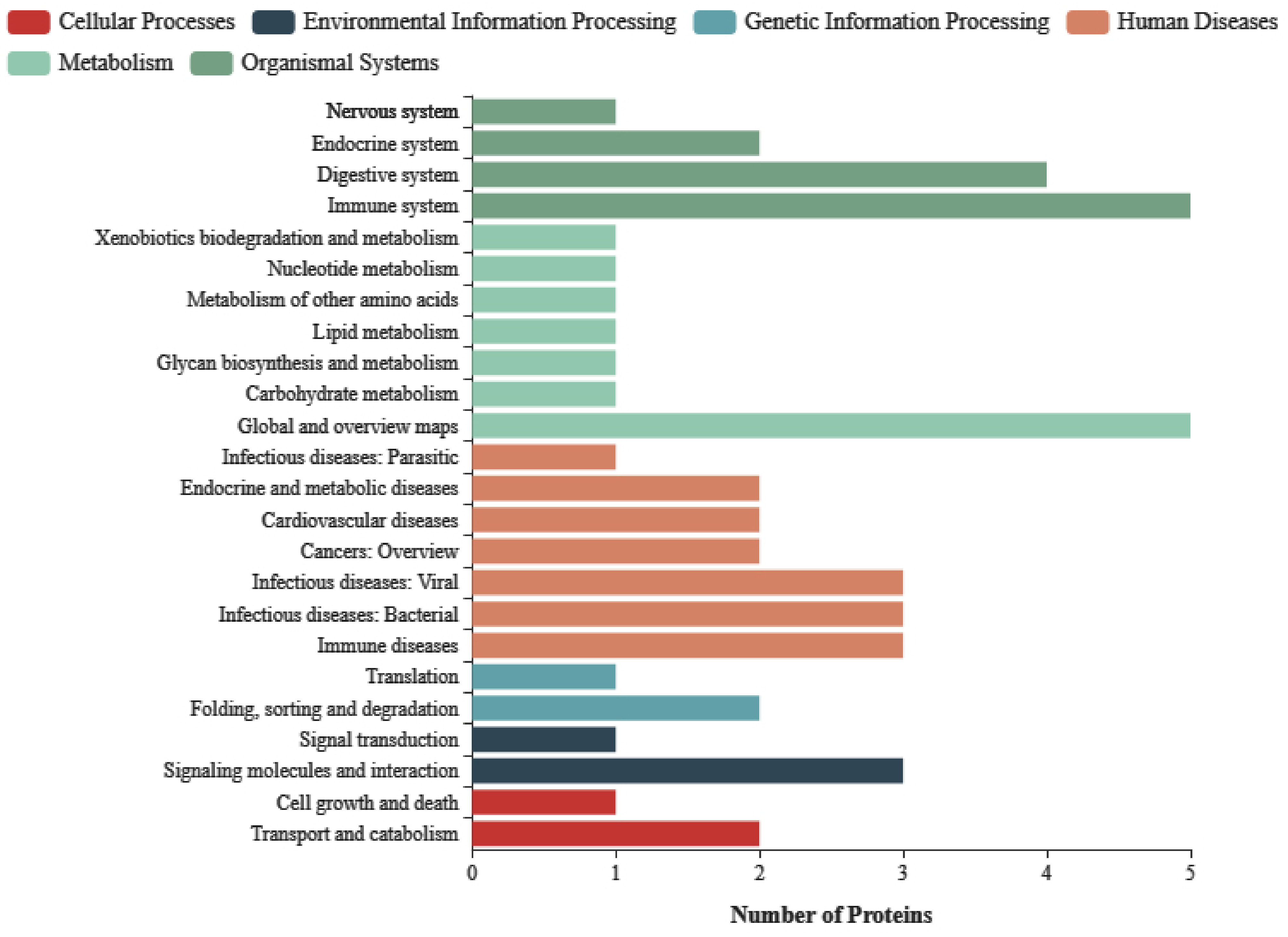

Cellular Processes
Environmental Information Processing
Genetic Information Processing
Human Diseases
Metabolism
Organismal Systems
Nervous system
Endocrine system
Digestive system
Immune system
Xenobiotics biodegradation and metabolism
Nucleotide metabolism
Metabolism of other amino acids
Lipid metabolism
Glycan biosynthesis and metabolism
Carbohydrate metabolism
Global and overview maps
Infectious diseases: Parasitic
Endocrine and metabolic diseases
Cardiovascular diseases
Cancers: Overview
Infectious diseases: Viral
Infectious diseases: Bacterial
Immune diseases
Translation
Folding, sorting and degradation
Signal transduction
Signaling molecules and interaction
Cell growth and death
Transport and catabolism
0
1
2
3
4
5
Number of Proteins
