# Supplementary figures and images for: HLA-A and HLA-DRB1 may play a unique role in ovarian teratoma-associated anti-N-methyl-D-aspartate receptor encephalitis
Source: Reprod Biol Endocrinol. 2020 Nov 7;18:107. doi: 10.1186/s12958-020-00661-5 (PMC7648266; doi:10.1186/s12958-020-00661-5)

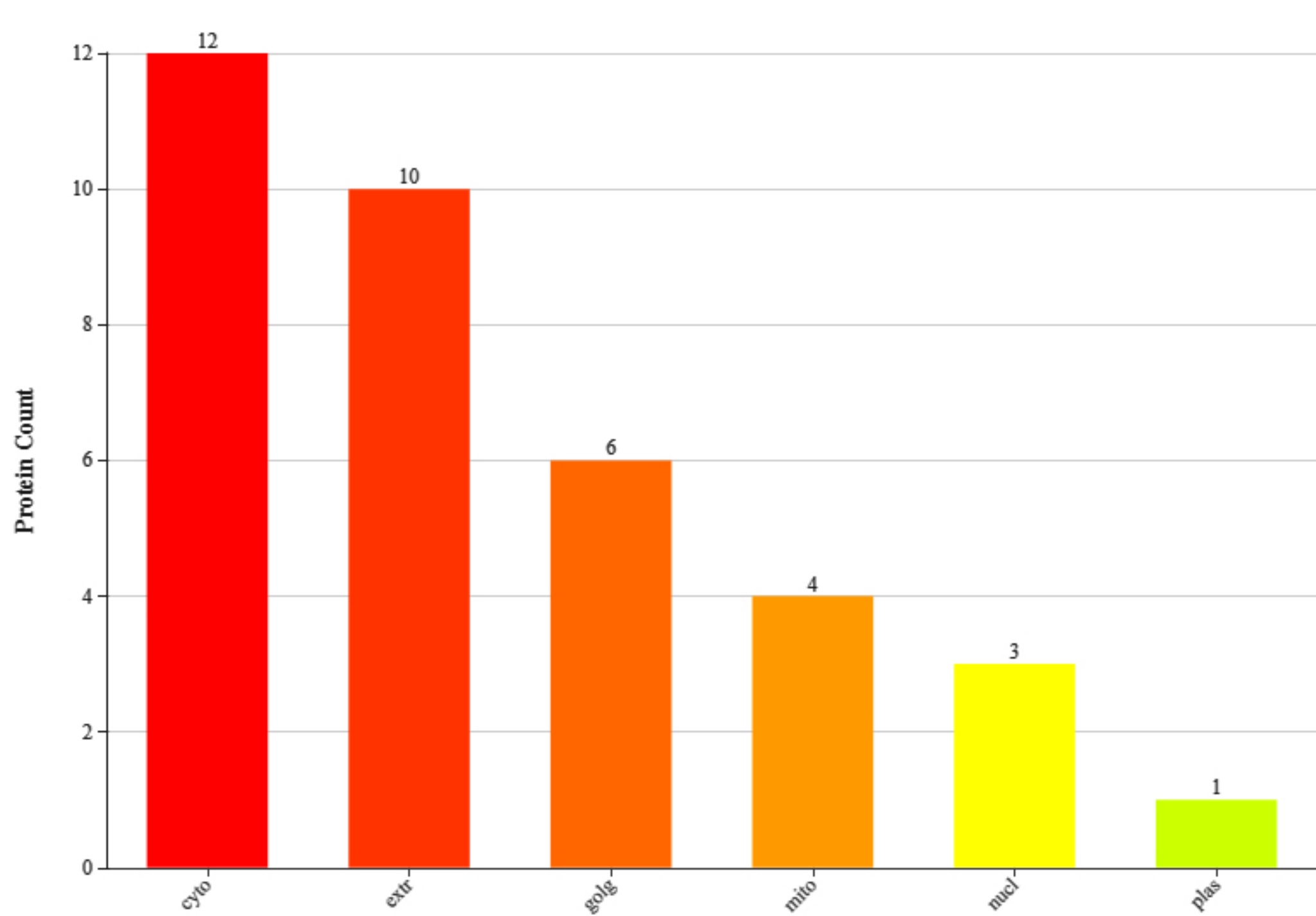

Supplement: Supplementary file 3 — Additional file 3: Supplementary Figure S2. Subcellular localization prediction (x-axis: subcellular structure; y-axis: protein count.) analysis of differentially expressed proteins. [file 12958_2020_661_MOESM3_ESM.pdf]

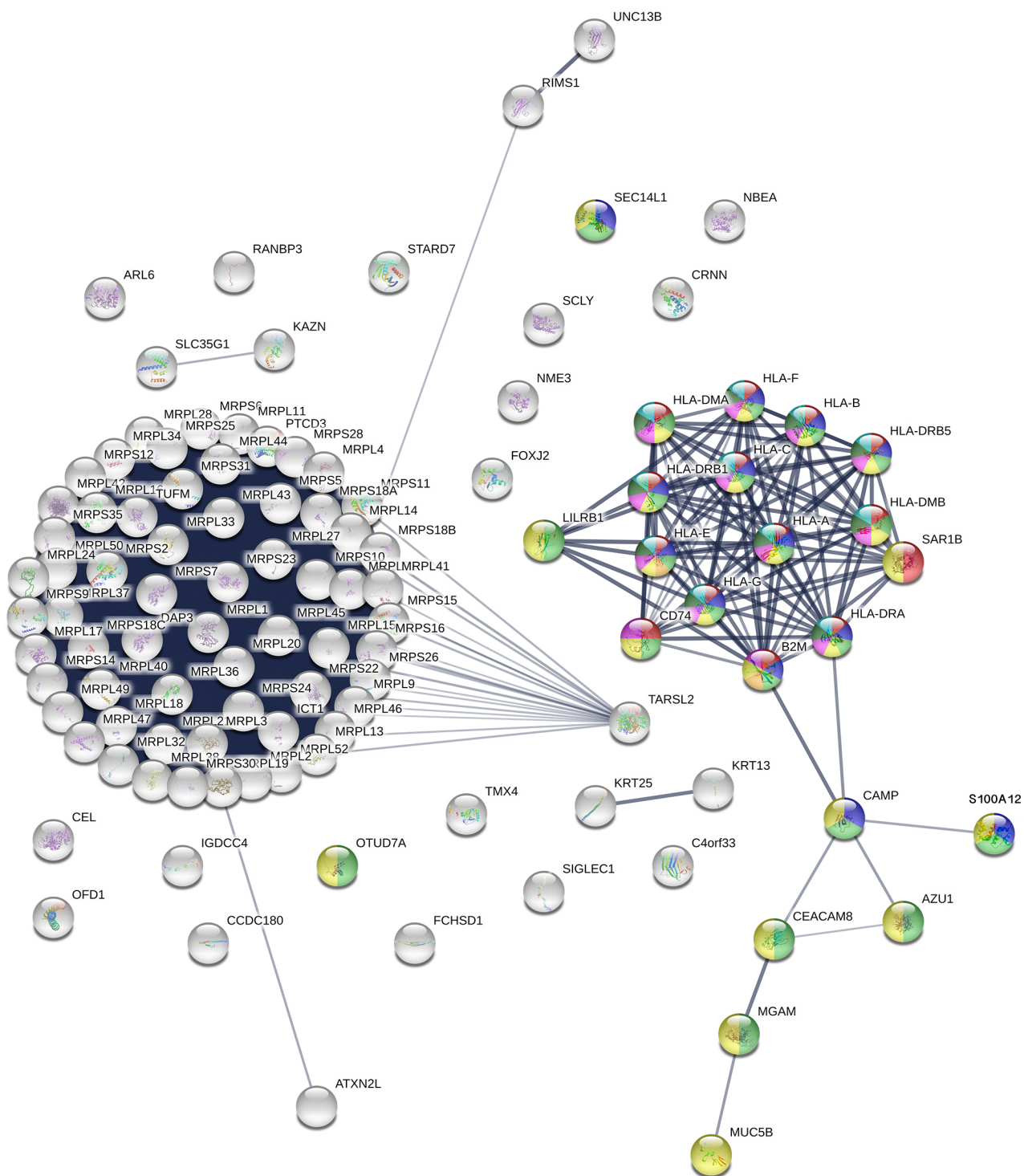

Supplement: Supplementary file 4 — Additional file 4: Supplementary Figure S3. Protein-protein interaction network of 36 differentially expressed proteins were subdivided into two categories: the network of colorful dot consisted of immune-related proteins. [file 12958_2020_661_MOESM4_ESM.pdf]

Actin

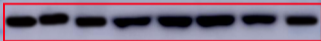

HLA-A

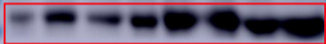

HLA-DRB1

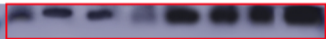

Supplement: Supplementary file 5 — Additional file 5: Supplementary Figure S4. Full-length western blots for Fig. 4a. [file 12958_2020_661_MOESM5_ESM.pdf]
